# Ticks, Human Babesiosis and Climate Change

**DOI:** 10.3390/pathogens10111430

**Published:** 2021-11-04

**Authors:** Jeremy S. Gray, Nicholas H. Ogden

**Affiliations:** 1 UCD School of Biology and Environmental Science, University College Dublin, D04 N2E5 Dublin, Ireland; 2 Public Health Risk Sciences Division, National Microbiology Laboratory, Public Health Agency of Canada, St-Hyacinthe, QC J2S 2M2, Canada; nicholas.ogden@canada.ca; 3 Groupe de Recherche en Épidémiologie des Zoonoses et Santé Publique (GREZOSP), Faculté de Medicine, Vétérinaire, Université de Montréal, St-Hyacinthe, QC J2S 2M2, Canada

**Keywords:** *Ixodes ricinus*, *Ixodes scapularis*, *Babesia microti*, *Babesia divergens*, climate, global warming

## Abstract

The effects of current and future global warming on the distribution and activity of the primary ixodid vectors of human babesiosis (caused by *Babesia divergens*, *B. venatorum* and *B. microti*) are discussed. There is clear evidence that the distributions of both *Ixodes ricinus*, the vector in Europe, and *I. scapularis* in North America have been impacted by the changing climate, with increasing temperatures resulting in the northwards expansion of tick populations and the occurrence of *I. ricinus* at higher altitudes. *Ixodes persulcatus*, which replaces *I. ricinus* in Eurasia and temperate Asia, is presumed to be the babesiosis vector in China and Japan, but this tick species has not yet been confirmed as the vector of either human or animal babesiosis. There is no definite evidence, as yet, of global warming having an effect on the occurrence of human babesiosis, but models suggest that it is only a matter of time before cases occur further north than they do at present.

## 1. Introduction

According to the 6th IPPC report, published in August 2021, global temperatures over the next 20 years are expected to “reach or exceed an average of 1.5 °C, unless there are immediate, rapid and large-scale reductions in greenhouse gas emissions”. Given that similar predictions were made, though with longer time scales, in each of the previous five reports, it now seems that such a global temperature increase is highly likely. This will result in an increasing number of heat waves, longer warm seasons and fundamental changes in rainfall patterns. Indeed, the first signs of these changes are already evident, most obviously in the natural world and relevant here in relation to arthropod vectors of disease [1]. It has been suggested that complex effects of climate change on both host communities and arthropod vectors could result in unanticipated spillover of pathogens from reservoir hosts into domesticated animals or humans resulting in disease emergence, depending on the host range of the pathogen [2], but the risk of emergence of novel *Babesia* spp. is unknown.

**Figure 1:**
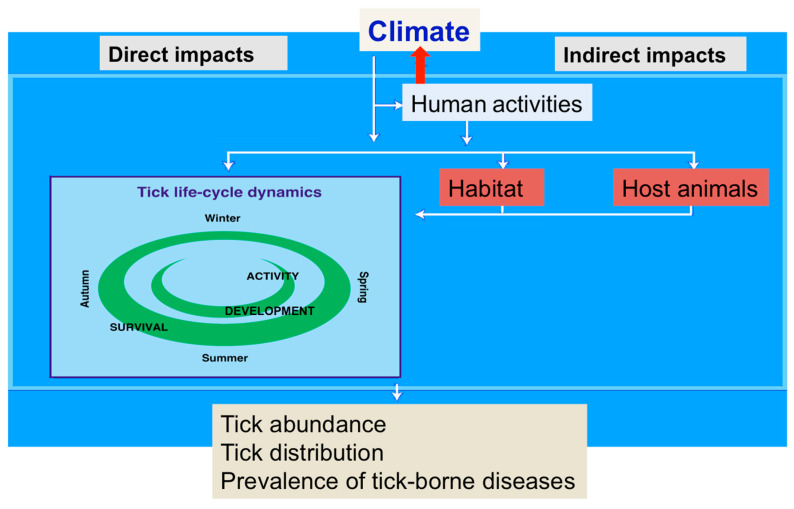
Factors determining the abundance and spread of *Ixodes* spp. Modified from Lindgren et al., 2000 [6].

The risk of human babesiosis can be affected by climate change in at least three different ways. Firstly, as poikilothermic organisms, the ixodid tick vectors of human babesiosis and the babesia pathogens within them can respond directly to changes in ambient conditions; secondly and more indirectly, both ticks and the vertebrate reservoirs of the pathogens can be affected by the impact of climate change on vegetation, resulting in changes to habitats (e.g., beech woods [3]), and to host food sources (e.g., masting events [4,5]), thirdly anthropogenic responses to climate change, notably human behaviour, but also the management of livestock reservoirs of infection, will affect exposure to the vectors and therefore the risk of disease (Figure 1).

The predominant vectors of human babesiosis are *Ixodes scapularis* transmitting *Babesia microti* in the USA, and *Ixodes ricinus*, transmitting *Babesia divergens* and *Babesia venatorum* in Europe [7]. *Babesia microti* also occurs in Europe, but human cases are extremely rare [8].

Several cases of *B. divergens* [9], *B. venatorum* [10] and *B. microti* [11], have been reported from China, and the vector, based on DNA detection, is suspected to be *Ixodes persulcatus* [11]. The same tick species is thought to be the vector of *B. microti* and an Asian lineage *B. divergens* in Japan [12,13]. Curiously, *I. persulcatus* has not been associated with either human or bovine babesiosis in Russia or Eastern Europe. Several other *Babesia* species in addition to *B. divergens*, *B. microti* and *B. venatorum* occasionally infect humans, but in most cases the identity of the vectors is unknown. The exceptions are *Babesia duncani*, which recent evidence suggests is transmitted by *Dermacentor albipictus* [14], a *Babesia crassa-*like parasite, probably transmitted by *I. persulcatus* or *Haemaphysalis concinna* [15], and an unnamed *Babesia* species in the USA, closely related to *B. divergens* and probably transmitted by *Ixodes dentatus*, a rabbit tick [16]. Since cases caused by these three *Babesia* species are rare, they will not be considered further here.

## 2. Life Cycles and Ecology of the Human Babesiosis Vectors

The three tick species responsible for most cases of human babesiosis, *Ixodes persulcatus*, *I. scapularis* and *I. ricinus* belong to the *Ixodes ricinus* species complex, consisting of at least another 15 species. They are three-host ticks, using separate hosts for each of the active stages, larva, nymph and adult, all of which engorge except for the male, which is probably not significantly involved in disease transmission. They are generalist species and feed on a very wide range of hosts, but there is some host selection, with larvae tending to feed preferentially on small mammals, nymphs on medium-sized mammals and birds, and adult females mainly on large mammals, such as deer and domestic livestock. However, there is a great deal of flexibility in these host preferences and large hosts can be heavily parasitised by the immature stages. Unlike most *Ixodes* species, which utilise hosts in nests and burrows, *I. persulcatus*, *I. ricinus* and *I. scapularis* attach to hosts in the open, using vegetation as ambush vantage points. When they have fed to repletion on their hosts over a few days, they drop off back into the vegetation, locate in the litter layer and commence development to the next stage, or commence egg development and then oviposition in the case of the female.

The life cycles of all three tick species are characterised by distinct seasonal activity of questing ticks, partly regulated by ambient conditions, so that little or no questing behaviour occurs at very high or very low temperatures, however, diapause is also a significant regulating mechanism. Diapause can be defined as a form of hormonally controlled arrested development or delayed behaviour that occurs prior to seasonally unfavourable environmental conditions. Conditioning of the ticks results from entrainment by certain environmental stimuli, particularly day length, and usually lasts for a set period. Laboratory studies have shown that temperature can affect diapause directly [17], but temperature may be more important in determining rates of tick development in relation to the seasonally-determined diapause conditioning periods. The role of diapause in regulating the life cycles of *I. persulcatus*, *I. ricinus* and *I. scapularis* has been reviewed recently [18].

The three *Ixodes* vectors of human babesiosis have very wide distributions encompassing several climate zones, for example *I. ricinus* occurs from the western seaboard of Europe to as far eastwards as the Ural Mountains and from the Atlas Mountains in North Africa to Northern Norway, though it is scarce in arid regions of southern Europe. The range of *I. persulcatus* is even greater, extending from Eastern Europe to the temperate Far East, and *I. scapularis* occurs from the southern states of the USA through the eastern seaboard as far north as southern Canada. Despite such wide ranges, the distribution of these tick species is limited by their susceptibility to desiccation when off the host. During development and especially when host-seeking (questing) they are exposed to ambient conditions and therefore confined to habitats that include humid microclimates (>80% RH) at the base of the vegetation, where the ticks obtain water by secreting a hygroscopic fluid onto their mouthparts and then ingesting it. Since questing may continue for several weeks, the ticks must make several journeys from the surface vegetation to soil level to replenish their water supply. The drier the atmosphere the more such trips, all costing energy, so that in hot, dry conditions survival may be limited. *I. persulcatus* differs from the other two species in that the immature stages are more reluctant to climb the vegetation and tend to quest in the litter layer [19], and southern strains of *I. scapularis* show similar behaviour relative to those from more northern regions in the USA [20], which may be a heritable adaptation to the drier conditions in the south. The consequence of the requirement of these ticks for humid microclimates when off the host is that their typical habitats tend to be woodlands with a substantial layer of vegetation litter. Deciduous and mixed forests offer the most favourable conditions, but coniferous forests may also harbour substantial numbers of ticks. Additionally, open habitats of rough vegetation such as the sheep-grazed uplands of north-western Europe, where maritime climates maintain mild winters and high humidity due to frequent rainfall, can maintain large numbers of ticks [21].

Another factor determining distribution and survival is temperature, which affects both development and questing, the lower thresholds of which probably vary with species, with regional differences occurring within tick species [22]. Cold air temperatures seem to have a limited effect on actual survival. For example, *I. scapularis* placed at −20 °C in the laboratory die rapidly, but engorged ticks placed in the litter layer in suitable woodland habitats in Canada over the winter (where air temperatures can often fall to less than −30 °C) have daily mortality rates no greater than those in summer, probably due to the insulating capacity of the litter layer (reviewed in Ogden et al. [23]). Similarly, it has been observed in Germany that *I. ricinus* populations are adversely affected by air temperatures of less than −15 °C only when the insulating snow cover is absent [24]. In the context of global warming, high temperatures are obviously important as drivers of desiccation in limiting tick survival, but laboratory studies suggest that even in the presence of high humidity, *I. ricinus* may suffer much higher mortality when temperatures exceed 30 °C [25].

The third vital component ensuring establishment and survival of tick populations is the availability of adequate numbers of appropriate hosts. In most habitats of the *Ixodes* species considered here, deer are essential hosts for the maintenance of the tick populations, because they are the only animals that feed significant numbers of adult female ticks, although in agricultural settings *I. ricinus* is also maintained by livestock, especially sheep and cattle [21]. Large hosts can feed all tick stages, but in woodland habitats small mammals and birds are important hosts of the immature stages, and many are essential components of tick-borne diseases, such as Lyme borreliosis, tick-borne encephalitis, several rickettsioses and human babesiosis caused by *B. microti.*

It is notable that ticks are increasingly recorded in urban areas and in such settings hedgehogs (*Erinaceus* spp.), another host that can feed all tick stages and thus maintain small populations of *I. ricinus*, could theoretically maintain zoonotic *B. microti* in the absence of large hosts [26,27]. As yet there are no reports of such foci, partly no doubt because zoonotic *B. microti* genotypes are rare in Europe [8].

## 3. The Roles of Reservoir Hosts of Human Babesiosis

*Babesia microti* is considered to be the most important cause of human babesiosis since it is responsible for the vast majority of cases, particularly in the USA. However, a study published in 2003 by Goethert and Telford [28], revealed that this is not a single species but consists of a complex belonging to three distinct clades utilizing a wide range of hosts, mostly rodents, but also shrews, dogs, foxes and raccoons. In the USA, some bird species were implicated in a single study as reservoirs of a *B. microti-*like organism [29]. However, the genotype involved is not known, and the distribution pattern of endemic areas of *B. microti-*babesiosis in the USA does not support long-distance distribution of the pathogen by birds. At present there is no evidence for significant bird involvement in the transmission of zoonotic *B. microti* genotypes, but this topic needs further study. In the Goethert and Telford study [28], most of the zoonotic genotypes turned out to belong to a single clade prevalent in the USA (though not confined to that country) and often referred to as the US-type or *B. microti* sensu stricto (s.s.), found in woodland mice, shrews and chipmunks. In Europe, very few cases of human babesiosis have been described despite widespread infection of rodents [8] and transmission by *I. ricinus* [30]. Although these cases appear to have been caused by rodent parasites, their rarity suggests that zoonotic genotypes are uncommon in Europe. In many regions the parasite is transmitted by *Ixodes trianguliceps*, which rarely bites humans, further reducing the risk of zoonotic babesiosis [8].

*B. venatorum* is a relatively recently described European zoonotic species [31], which has since been reported to have caused many more cases in China [10]. In Europe, good evidence now exists that the reservoir host of *B. venatorum* is roe deer (*Capreolus capreolus*) [32,33]. Sika deer (*Cervus nippon*) probably fulfils this role in China. Thus, two zoonotic *Babesia* spp. (*B. microti* s.s. and *B. venatorum*) are firmly associated with woodland. The third species, *B. divergens*, has until recently been considered to be an exclusive cattle parasite. With the advent of molecular taxonomy this parasite has also been reported from red deer (*Cervus elaphus*) and roe deer (*C. capreolus*) [34], but there is no evidence for wild deer as a source of infection for cattle or vice versa, although splenectomised red and roe deer can evidently be infected with *B. divergens* from cattle [35]. Current data suggest that almost all isolates from human cases closely match bovine babesia sequences, only two with less than 99.9% 18S rRNA gene homology, and having little identity with babesia sequences from deer [8]. The host origins of these babesias are unknown. It must be concluded that at present there is no evidence for deer as a source of *B. divergens* infection of humans and that human cases are predominantly associated with cattle and thus with agricultural rather than woodland habitats.

## 4. Expected Impacts of Climate Change on the Vectors

While in general, warming temperatures are likely to make northern regions more hospitable for ticks, and possibly less so closer to the equator, direct effects on tick survival of increasing temperatures and changes in rainfall patterns on tick survival in many regions of the northern hemisphere may be limited, because of the protection afforded by the typical woodland habitats and also by their ability to undergo developmental and behavioural diapause to avoid unfavourable conditions [18].

Of greater impact on tick population survival is the expected effect of warming temperatures on rates of development from one life stage to the next, and on host-seeking activity. Because the duration of development from one life stage to the next is mostly temperature-dependent (within the constraints of diapause), warmer temperatures will probably mean shorter tick life cycles, and shorter development times will probably be coupled with extended periods of the year when temperatures are suitable for tick activity [36]. Laboratory experiments by Gilbert et al. [22] also suggest that a greater proportion of *I. ricinus* in the questing phase will become active as temperatures increase, and the interaction of temperature with humidity, driving the saturation deficit, also directly impacts host-seeking activity [37]. The success of host seeking can therefore be influenced directly by temperature effects on the ticks, but is also determined by the abundance and activity of hosts, which will be affected by the temperature-dependent availability of forage.

## 5. Projected Effects of Climate Change on Tick and *Babesia* spp. Distributions

**Figure 2:**
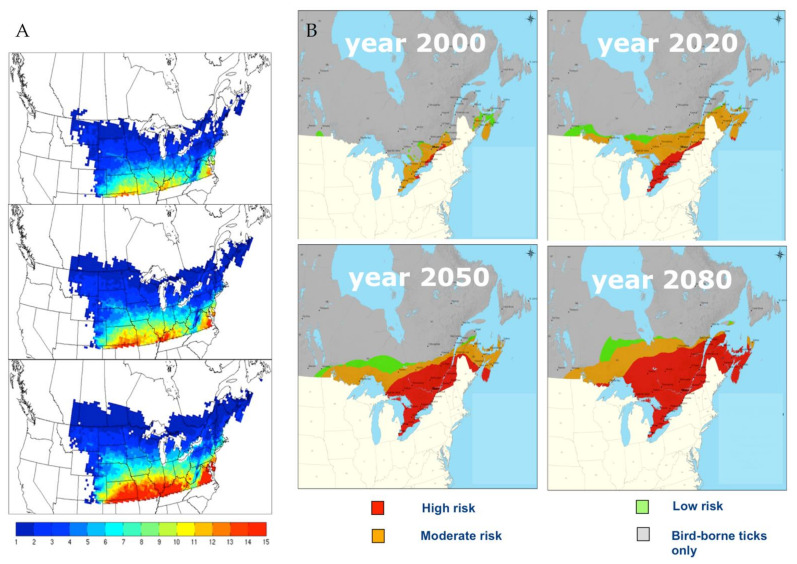
(**A**) Maps of values of the basic reproduction number (*R*_0_) of *Ixodes scapularis* in North America, estimated from ANUSPLIN observed temperature (1971–2000: upper panel), and projected climate obtained from the climate model CRCM4.2.3 following the SRES A2 greenhouse gas emission scenario for 2011–2040 (middle panel) and 2041–2070 (bottom panel). The colour scale indicates *R*_0_ values. Temperature conditions that result in an *R*_0_ of >1 permit survival of *I. scapularis* populations. Reproduced from Ogden et al., 2014 [38]. (**B**) Risk maps for the occurrence of *Ixodes scapularis* in Canada in response to increasing temperatures associated with climate change. The methods used to generate these maps are described by Ogden et al., 2008 [40].

With the future temperatures projected by climate models, it is expected that the northern limit of the range of *I. scapularis* will expand northwards [38,39] and the leading edge of this expansion is now north of the Canadian border (Figure 2).

**Figure 3:**
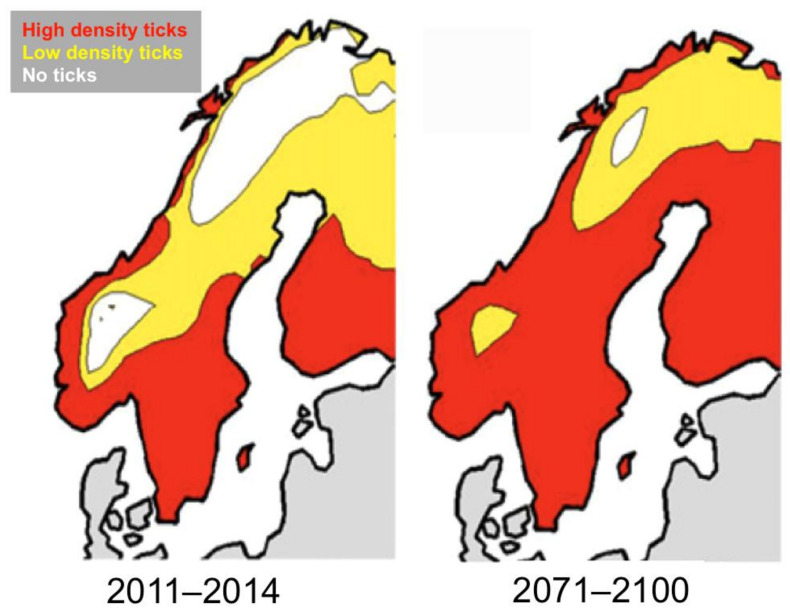
Climate change prediction of *Ixodes ricinus* distribution in Scandinavia based on the length of the vegetation growth period, IPCC 2000 high emission scenario. Modified with permission from Jaenson and Lindgren, 2011 [41].

Several studies in Europe have predicted a northwards expansion of the geographic range of *I. ricinus* [41,42,43], (for example see Figure 3).

The models of Porretta et al. [42] and Alkishe et al. [43] also suggest that the distribution of *I. ricinus* is likely to extend eastwards, into habitat currently occupied by *I. persulcatus.* Additionally, *I. ricinus* is predicted to occur at increasingly higher altitudes in mountainous regions [44]. For the main tick vectors of *Babesia* spp. from the northern hemisphere, range expansion driven by climate would only be possible where suitable habitats occur. However, these tick species are, for the most part, woodland habitat generalists and as long as woodland habitats occur, it is likely that the ticks will survive in at least some of them, providing the woodlands also support host densities that are high enough. Some studies have suggested that southern range limits of ticks may contract northwards as more southern regions become too hot for ticks, particularly due to high temperatures inhibiting host-seeking tick activity [25,45]. Increased climate variability and extreme weather events (extreme heat and rainfall) may have relatively limited positive or negative impact on the ticks (compared to dipteran vectors) because of their relatively long multi-year life cycles and the capacity of their woodland habitats to provide an environment that protects the ticks from extreme weather [46]. Impacts of climate change on geographic ranges of hosts such as the white-footed mouse, *Peromyscus leucopus*, will likely have impacts on the geographic ranges and level of entomological risk of *B. microti* in current endemic areas [47]. It is possible that efficient host-to-tick transmission only occurs for a short period after initial infection [48] and if so, locations where there is seasonally synchronous activity of nymphal ticks (that infect the mice) and larval ticks (that acquire infection from mice), may pose a high risk. Effects of climate change on tick development and activity may cause changes to tick seasonality, resulting in locations where synchronous seasonal immature tick activity produce *B. microti* hot spots [23]. In addition to effects on synchrony, increased temperatures may result in changes in the proportions of the tick population feeding at different times of the year. Such an effect was observed in 1976 and 1977 in Ireland when an unusually hot summer in 1976 caused early activity of summer larvae, resulting in a marked increase in the proportion of nymphs active in the late autumn that year and the following spring [49], providing an indication of possible future effects of global warming.

Ticks themselves have very limited capacity for dispersal, and for any change in geographic range to occur, ticks and tick-borne pathogens including *Babesia* spp. need to be dispersed by ticks. Evidence suggests that two processes may be at play [50]—local dispersal that is likely by terrestrial hosts and breeding birds [51], and long-range dispersal by ticks carried on passerines that carry ticks northward in spring [40]. Some of these ticks may be infected with *B. microti*, but it is likely that dispersal by migratory birds is inefficient for *B. microti,* because this pathogen is not transmitted vertically by ticks (transovarial transmission) [30], and birds have, as yet, not been confirmed as reservoirs. As any larvae feeding on migratory birds would not be infected before or during their dispersal by birds, the only infected ticks that birds might carry would be nymphs infected as larvae on small mammal reservoir hosts, as demonstrated by the study of Wilhelmsson et al. [52]. Adult female ticks arising from such nymphs are likely to feed on deer rather than *B. microti* reservoir hosts and would pose little zoonotic risk since they would probably be free of infection following their second moult [30].

## 6. Observed Effects of Climate Changes

### 6.1. Climate Effects on Ticks

Studies in Canada suggest that the northern extent of the range of *I. scapularis* is determined by the limit of temperature conditions that allow ticks to complete their life cycles; i.e., when it is probable that an engorged, mated female tick gives rise to at least one other engorged, mated female tick (using the definition of Anderson and May [53] for a macroparasite, when the basic reproduction number of the tick is ≥1).

**Figure 4:**
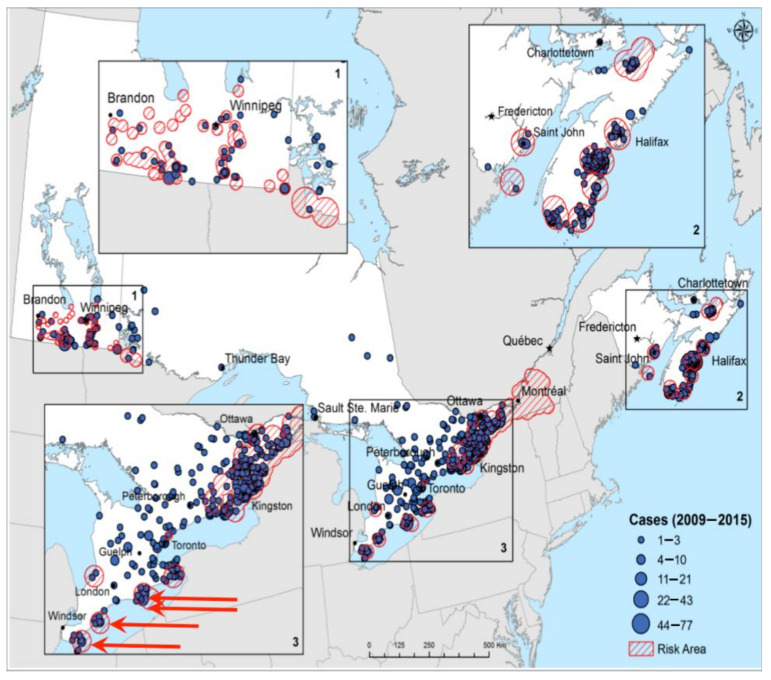
Surveillance for *Ixodes scapularis* populations in central and eastern Canada conducted from 2009 to 2015. Regions where *I. scapularis* populations have been identified by field surveillance are shown as red hatched areas. In 2004 there were only four known *I. scapularis* populations in locations shown by the red arrows. Tick populations have been identified in surveillance programs for Lyme disease (blue circles show municipalities where human Lyme disease cases have been identified). Infections due to *Babesia microti* are not yet nationally notifiable (reproduced with permission from Gasmi et al., 2017 [57]).

Combinations of data from active field surveillance for ticks, passive tick surveillance (involving detection of ticks at medical and veterinary clinics and by the public), and by inference from surveillance for human cases of tick-borne disease, such as Lyme borreliosis, have detected northern expansion of the range of *I. scapularis* [54,55,56] (Figure 4).

Figure 4 shows that *I. scapularis* was only detected at four locations in 2004 (red arrows), but that between 2009 and 2015 ticks and Lyme borreliosis cases had emerged in many other places. Surveillance data have detected a spatio-temporal pattern of range expansion of *I. scapularis* that is consistent with a warmer climate being a key determinant of range spread, and that support the accuracy of model-derived temperature thresholds for *I. scapularis* population survival (reviewed in Ogden et al. [23]). Furthermore, expansion of the tick range has occurred during a period of warming that is now considered a climate anomaly associated with anthropogenic climate change.

Similar trends have been observed for *I. ricinus*, particularly in Scandinavia [6,58,59], but also in Russia [60]. Additionally, the predicted altitudinal changes [23] in *I. ricinus* distribution have already been reported. In 1979 ticks were found up to 700 m a.s.l. in mountainous regions of the Czech Republic, but in 2002 were collected at 1100 m [61,62], and more recently (2020) have been found higher still at 1700 m in the Italian Alps [63]. An earlier study in 1993 had shown that *I. ricinus* was unable to complete its life cycle at such altitudes in the Czech Republic [64].

**Figure 5:**
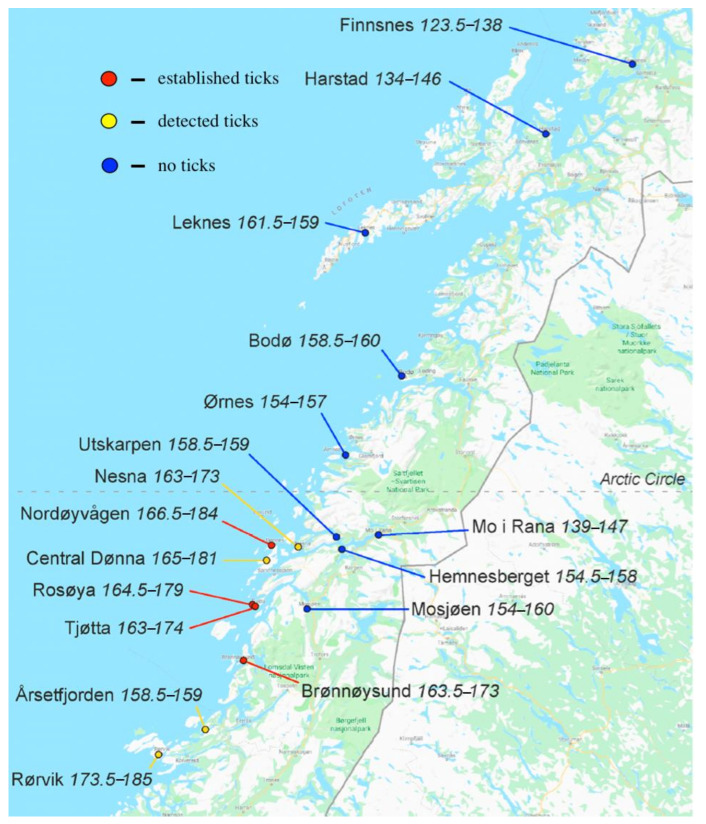
Vegetation growing season length (VGSL) in days in 1961–1990 and 1991–2015 correlated with the presence of *Ixodes ricinus* in northern Norway. The VGSL threshold for tick establishment was estimated to be approximately 170 days. Modified with permission from Hvidsten et al., 2020 [59].

In Norway, Hvidsten et al. conducted one of the few surveys based on direct observation of *I. ricinus* occurrence at the northern limits of its distribution, collecting specimens by drag-sampling, small mammal trapping, from domestic animals, mainly dogs, and by mailed submissions [59]. Attempts were made to differentiate locations where *I. ricinus* populations were established from those where a few adventitious ticks had been observed or no ticks were detected. The criteria for tick establishment in a local region were the presence of all three life cycle stages in two successive years [65]. Estimates of the vegetation growing season length (VGSL), defined as the number of days when the mean temperature exceeds 5 °C, suggest that established tick populations occurred where the VGSL exceeded 170 days (Figure 5).

The period of 170 days VGSL is the same minimum value for tick establishment estimated for Scandinavia by Jaenson and Lindgren [41] and was the basis for their projections of tick distribution changes over the next few decades (Figure 3). When the average VGSL values in the Hvidsten et al. study for the period 1961–1990 are compared with those for 1991–2015, an increase in VGSL is evident at all locations, clearly associating the expanding tick distribution with rising temperatures. The most likely mechanism for this temperature effect is the time required for each tick stage to complete development within a season, as demonstrated by Daniel [64] in his altitude study, but low temperatures will also limit questing activity, and Gilbert et al. [22] have shown that *I. ricinus* nymphs from higher latitudes can quest at lower temperatures than those from more southerly regions.

### 6.2. Climate Effects on Reservoir Hosts

In addition to direct effects on ticks, rising temperatures will also affect their hosts, which is particularly important when these hosts serve as reservoir hosts for tick-borne pathogens. In the case of *B. divergens*, the pathogen’s distribution is closely associated with that of cattle. Infections appear to result from local transmission by established tick populations [66], and since tick populations are expanding northwards, it is not surprising that there is some evidence, though indirect, for bovine babesiosis in more northern locations than in previous decades [67]. Since *B. divergens* is transmitted transovarially, infected larval or nymphal ticks could be deposited by birds far to the north of established tick populations. However, there is very little evidence for infections transmitted to cattle by such adventitious ticks, with only one suspected case in the far north of Norway occurring in the last 20 years [66]. A more definite climate effect on bovine babesiosis, though on a local scale, occurred recently in the south of England in February 2019, when temperatures exceeded the average for the time of year by more than 10 °C, causing very early tick activity and an outbreak of bovine babesiosis involving 20 cattle [68]. *Babesia venatorum* is also transmitted transovarially and is associated with roe deer, so presumably the distribution of this pathogen has been affected by the northward range expansion of its host [58]. It can also be distributed by birds carrying infected ticks, which led to speculation that its detection in sheep in Scotland may have resulted from the deposition of ticks by migratory birds from Norway [69]. At present there are insufficient data on the distribution of *B. venatorum* to associate it with any climate change effect.

Genotypes of *B. microti* have been detected in many vertebrate species but those that cause human babesiosis in north-eastern North America appear to be limited to the white-footed mouse, *Peromyscus leucopus*, short tailed shrews, *Blarina* spp., and chipmunks, *Tamia striatus*. Since, in the absence of transovarial transmission, neither birds nor deer can play a significant part in the introduction of the parasite to new reservoir host populations, and it appears that migration of infected small mammals is the main means by which the pathogen can emerge in new areas. *B. microti* infections therefore lag well behind the spread of other *I. scapularis*-borne diseases such as Lyme borreliosis and human granulocytic anaplasmosis [70], both of which can infect birds, though *B. microti* infections are spreading within the northeast and upper midwest endemic regions [50,71]. While the tick vector has spread north into Canada, human babesiosis remains exceedingly rare and to date only three cases of autochthonous infection have been recorded there [72].

There is little information on the effects of climate change on the small mammal reservoir hosts of *B. microti*, but intuitively one might expect milder winters to result in their improved survival, driving expansion of their populations. In *P. leucopus*, one of the main reservoir hosts, tick transmission is relatively inefficient, but appears to be facilitated by the agent of Lyme disease, *B. burgdorferi* sensu stricto [73], which is now prevalent in southern Canada and for which *P. leucopus* is also an important reservoir host. Furthermore, the persistence of *B. microti* in its rodent hosts, *Microtus* spp. and *P. leucopus*, is enhanced by vertical transmission [74,75], so range expansion of these rodents is likely to be fundamental to the spread of *B. microti.* Roy-Dufresne et al. [76] used an ecological niche factor analysis to study the potential effect of global warming on the distribution of *P. leucopus* and concluded that by 2050 the range of this rodent species could have expanded northwards by 3° latitude. Considering that the upper midwest *B. microti* endemic area is only just across the US border, it seems likely that significant numbers of cases will eventually occur in Canada. Indeed, the three recorded autochthonous Canadian *B. microti* infections were apparently all acquired in southern Manitoba not very far from the endemic region in Minnesota [72], although unfortunately it is not known whether the same genotypes responsible for *B. microti* human babesiosis in the US were involved in these Canadian cases. An alternative explanation for the appearance of human babesiosis in new areas, is that zoonotic genotypes of *B. microti* occur in the absence of *I. scapularis*, being transmitted by tick species that generally do not bite humans, for example *Ixodes angustus.* While such cryptic cycles exist [77], there is little evidence so far that they have played a part in the spread of zoonotic babesiosis caused by *B. microti*, although they might have had a role in the establishment of the two separate foci in the northeast and upper midwest of the US, in which the *B. microti* genotypes show distinct differences from each other [78]. *Ixodes scapularis* (or *I. dammini*) is thought to have spread from coastal refugia in the 1950s as a result of reforestation and the growing deer population [79], and it is possible that in the upper midwest *B. microti* genotypes maintained in cryptic cycles were then able to infect this newly arrived bridge vector and thus establish a new focus of human babesiosis.

## 7. Conclusions

While observations suggest that tick populations have been responding to increasing temperatures with a northwards expansion for some years, it is not possible at present to be certain that the occurrence of human babesiosis has been affected by climate change. This is partly because of lack of data, particularly in Europe, where human babesiosis is much rarer than in North America, but also because the distributions of the pathogens involved depend on infected reservoir hosts, in addition to ticks, and the factors affecting the movements of these animals (small mammals, deer and domestic cattle) are influenced by other factors in addition to climate, notably landscape changes resulting from anthropocentric activity. Nevertheless, observations and models suggest that it is only a matter of time before human babesiosis cases occur more frequently, out of season and further north than at present as a result of climate change.

## Data Availability

Not applicable.
